# Description of two new species of *Tonsilla* Wang & Yin, 1992 with an updated key to species (Araneae, Agelenidae)

**DOI:** 10.3897/zookeys.944.48575

**Published:** 2020-06-30

**Authors:** Ke-ke Liu, Hui-pu Luo, Xiang Xu, Zhiwu Chen, Yong-hong Xiao

**Affiliations:** 1 College of Life Science, Jinggangshan University, Ji’an 343009, Jiangxi, China; 2 College of Life Science, Hunan Normal University, Changsha 410081, Hunan, China; 3 The National & Local Joint Engineering Laboratory of Animal Peptide Drug Development (Hunan Normal University), National Development and Reform Commission, Changsha 410081, China

**Keywords:** Jiangxi, Coelotinae, identification key, Jinggang Mountain, spider, taxonomy

## Abstract

Two new species of *Tonsilla* Wang & Yin, 1992 are described from Jinggang Mountain National Nature Reserve, Jiangxi Province, China: *T.jinggangensis* K. Liu & X. Xu, **sp. nov.** (♀) and *T.subyanlingensis* K. Liu & X. Xu, **sp. nov.** (♂♀). The new species are illustrated, and their distributions are mapped. Detailed generic characters and an updated key to *Tonsilla* species are also given.

## Introduction

At present, the Coelotinae, with approximately 770 species belonging to 33 genera, is the largest subfamily of Agelenidae. The number of species in this subfamily has increased in the last five years greatly due to more than 20 publications, so that now the Agelenidae is the tenth largest spider family (WSC 2020). More than half of the species of Coelotinae belong to 24 genera and are known from China (WSC 2020); therefore, China has most species- and genus-rich fauna in the world. However actual species richness in the region remains unrevealed.

*Tonsilla* Wang & Yin, 1992 is relatively small genus with 11 named species which are known exclusively from China. It is a relatively well-studied genus due to numerous publications (see WSC 2020), although half of the species are known by a single sex only (five by females and one by a male). Only one species was described from Jiangxi Province (Zhu et al. 2017).

While studying the Agelindae from the Jinggang Mountain National Nature Reserve, Jiangxi Province, we found two new species belonging to *Tonsilla*, and the main goals of this paper are, therefore, to describe these new species, to provide a key to all species of the genus, and to discuss the affinities of *Tonsillia*.

## Materials and methods

Specimens were examined using a Zeiss Stereo Discovery V12 stereomicroscope with a Zoom Microscope System. Both the male palps and female copulatory organs were detached and examined in 75−80% ethanol under a Zeiss Axio Scope A1 compound microscope with a KUY NICE CCD. For SEM photographs, the specimens were dried on filter paper and photographed with the ZEISS EVO LS15 scanning electron microscopes under a low vacuum. The specimens were subsequently stored in 75% ethanol after SEM.

All measurements were made by using ImageView CM2000 software and in millimetres. Leg measurements are given as total length (femur, patella, tibia, metatarsus, tarsus). All the specimens are deposited in the Animal Specimen Museum, Life Science of College, Jinggangshan University (ASM-JGSU).

Terminology of the male and female copulatory organs follows Wang (2003) and Wang et al. (2010). Leg spines are documented by dividing each leg segment into three aspects, dorsal and ventral, the latter being divided into prolateral and retrolateral, e.g., I femur 0 (dorsal) 2 (prolateral ventral) 2 (retrolateral ventral); I tibia 1 (dorsal) 4 (prolateral ventral) 4 (retrolateral ventral). An asterisk (*) indicates a slender spine. The abbreviations used in the text and figures are:

**Eyes**

**ALE** anterior lateral eye;

**AME** anterior median eye;

**PLE** posterior lateral eye;

**PME** posterior median eye;

**Male palp**

**BLC** basal lamella of conductor;

**CF** cymbial furrow;

**Con** conductor;

**DAC** dorsal apophysis of conductor;

**Em** embolus;

**MA** median apophysis;

**PA** patellar apophysis;

**RTA** retrolateral tibial apophysis;

**VTA** ventrolateral tibial apophysis;

**Epygine**

**At** atrium;

**CD** copulatory duct;

**CO** copulatory opening;

**EH** epigynal hood;

**ET** epigynal teeth;

**FD** fertilization duct;

**SH** spermathecal head;

**Spe** spermatheca;

**Legs**

**fe** femur;

**mt** metatarsus;

**pa** patella;

**ta** tarsus;

**ti** tibia.

## Taxonomy

### Family Agelenidae C.L. Koch, 1837

**Subfamily Coelotinae F.O. Pickard-Cambridge, 1893**

#### Genus Tonsilla

Taxon classificationAnimaliaAraneaeAgelenidae

Wang & Yin, 1992

CAAF1961-B3F2-5406-AA85-215F17D7C924

Tonsilla Wang and Yin 1992: 263.
Tonsilla : Wang 2003: 569.
Tonsilla : Yin et al. 2012: 1029.
Tonsilla : Zhu et al. 2017: 547.

##### Type species.

*Tonsillatruculenta* Wang & Yin, 1992.

##### Diagnosis.

**Figure 1.**
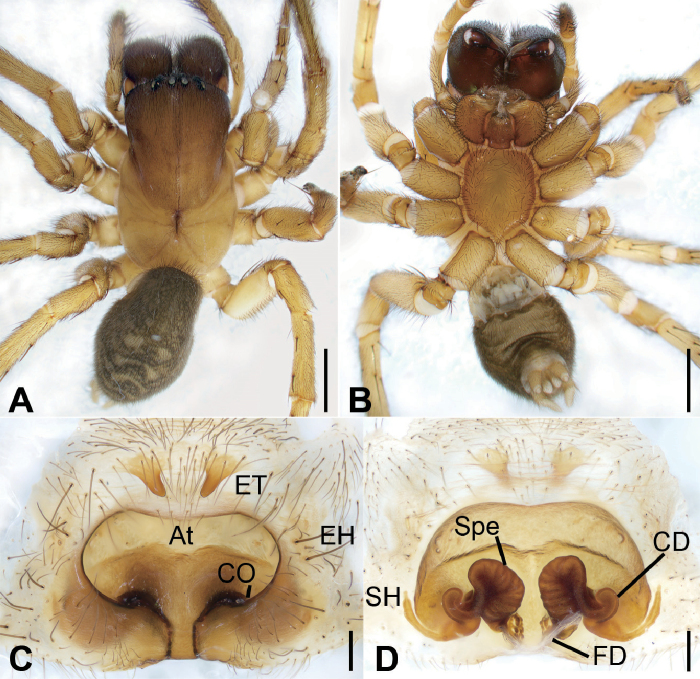
*Tonsillajinggangensis* sp. nov., female holotype **A** habitus, dorsal view **B** same, ventral view **C** epigyne, ventral view **D** vulva, dorsal view. Scale bars: 2 mm (**A, B**), 0.2 mm (**C, D**). Abbreviations: At – atrium, CD – copulatory duct, CO – copulatory opening, EH – epigynal hood, ET – epigynal teeth, FD – fertilization ducts, SH – spermathecal heads, Spe – spermathecae.

**Figure 2.**
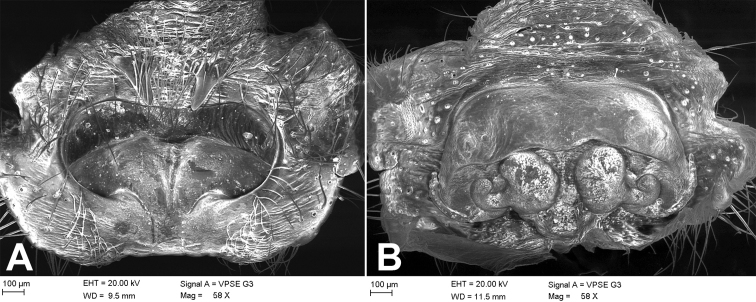
SEM images of *Tonsillajinggangensis* sp. nov., female holotype **A** epigyne, dorsal view **B** vulva, ventral view.

Males of this genus can be easily distinguished from these of other genera of Coelotinae by the male palpal patella with a large strong apophysis, which is more than half of the patella length (Figs 5D, E, 7F) (vs small, less than half length of palpal patella in other genera) and conductor with dorsal apophysis (Figs 5C–E, 7A–C, E) (vs without dorsal apophysis). Females of *Tonsilla* are most similar to those of *Pireneitega* in having the large epigynal atrium and large copulatory ducts, and easily differentiated from them by the sub-spherical spermathecae (Figs 1D, 2B, 6D, 7I) (vs strongly convoluted) and from other genera by epigynal teeth located on the anterior atrial margin close to each other (Figs 1C, D, 2A, B, 6C, D, 7H, I) (vs widely separated epigynal teeth located bilaterally in other genera).

##### Description.

***Body size*** 7.0–17.0 mm. The morphological appearance of this genus is similar to that of other coelotines. Carapace anteriorly narrowed to between 0.6 and 0.9 times its maximum width. PLE–PLE covered half width of anterior carapace. Chelicerae (Figs 1B, 6B) robust, as wide as half of carapace, with long fang, usually with 3 promarginal and 2 or 3 retromarginal teeth. Endites (Figs 1B, 6B): bean-shaped, longer than wide, with a relatively narrow base, ectal margins distinctly convex; ectal edge concave. Labium: longer than wide, posteriorly narrowed. Sternum (Figs 1B, 6B): longer than wide, shield-shaped, almost straight anteriorly, with slightly convex sides, and pointed posteriorly.

***Male palp*** (Figs 5C–E, 7A–G): patella with large apophysis, more than half of the patella length, strongly sclerotized, extending to dorsal part of patella; tibia with 2 apophyses, ventroretrolateral and retrolateral, the former broad, arising basally, extending along the retrolateral margin, anteriorly with slightly protruding beyond the distal or subdistal part of tibia, with widely truncated tip, the later from small to large, arising latero-medially; cymbium length/width ratio varies 1.8–2.4 in dorsal view, cymbial furrow less than half of cymbial length, in *T.defossa* and *T.subyanlingensis* sp. nov. from half to more than half of cymbial length; conductor long, anterior part with a distinct furrow or without, with a bifurcated tip or not, with a fine dorsal apophysis of conductor arising from its base; embolus flat and thin, arising at 6 o’clock position, with broad basally, roundly bent and coiled; tegular apophysis spoon-like.

***Epigyne***: atrium from large to small, heart-shaped, posteromedially located, broad and anteriorly located in *T.defossa*, with an arch-shaped or triangular septum arising antero-medially in *T.truculenta* Wang & Yin, 1992; copulatory openings located postero-laterally in the atrium; epigynal teeth tube-shaped or horn-like, flattened in *T.subyanlingensis* sp. nov., located antero-medially, separated by its length or less, or slightly fused basally; copulatory ducts sac-shaped, mostly rounded, tube-shaped in *T.jinggangensis* sp. nov., *T.subyanlingensis* sp. nov., and *T.yanlingensis*; spermathecae spherical or ovoid, duct-shaped in *T.defossa*, widely separated or close to each other; spermathecal heads arising anteriorly or posteriorly, from short or long; fertilization ducts arising from the posterior part of spermathecae.

##### Distribution and habitat.

The genus is known from subtropics in south China (Sichuan, Anhui, Guizhou, and Jiangxi provinces). Habitats of these spiders are not very diverse, usually found in woody debris, among tree roots on the ground, in humus, and under stones or tree bark.

##### Composition.

*T.defossa* Xu & Li, 2006 (♂♀; Sichuan), *T.distalis* Zhang, Zhu & Wang, 2017 (♀; Guizhou), *T.eburniformis* Wang & Yin, 1992 (♀; Hubei), *T.jinggangensis* K. Liu & X. Xu, sp. nov. (♀; Jiangxi), *T.lyrata* (Wang, Yin, Peng & Xie, 1990) (♀; Hunan), *T.makros* Wang, 2003 (♂; Guizhou), *T.mopanensis* Zhang, Zhu & Wang, 2017 (♂♀; Guizhou), *T.rostrum* Jiang, Chen & Zhang, 2018 (♂♀; Guizhou), *T.subyanlingensis* K. Liu & X. Xu, sp. nov. (♂♀; Jiangxi), *T.tautispina* (Wang, Yin, Peng & Xie, 1990) (♀; Jiangxi), *T.truculenta* Wang & Yin, 1992 (♂♀; Hunan, Hubei, Guizhou, Sichuan), *T.variegata* (Wang, Yin, Peng & Xie, 1990) (♂♀; Anhui), and *T.yanlingensis* (♀; Hunan).

### Key to species of *Tonsilla*

**Males** (males of *T.distalis*, *T.eburniformis*, *T.jinggangensis* sp. nov., *T.lyrata*, *T.tautispina* and *T.yanlingensis* are unknown)

**Table:** 

1	Cymbial furrow less than half of cymbial length (see Jiang et al. 2018: fig. 24C)	**2**
–	Cymbial furrow more than half of the cymbial length (Fig. 5E)	**4**
2	Patellar apophysis shorter than patella (see Zhu et al. 2017: fig. 359E)	**3**
–	Patellar apophysis as long or longer than patella (see Zhu et al. 2017: fig. 358C)	**6**
3	Ventrolateral tibial apophysis not extending beyond the distal end of tibia (see Zhu et al. 2017: fig. 353C–E)	***T.defossa***
–	Ventrolateral tibial apophysis extending beyond the distal end of tibia (Fig. 5D, E)	**4**
4	Retrolateral tibial apophysis large and strong, longer than half of tibia (Figs 5D, E, 7B, E, F)	***T.subyanlingensis* sp. nov.**
–	Retrolateral tibial apophysis small, less than half length of tibia (see Zhu et al. 2017: fig. 362D)	**5**
5	Retrolateral tibial apophysis arising from the base of tibia (see Zhu et al. 2017: fig. 358C)	***T.makros***
–	Retrolateral tibial apophysis arising from the middle part of tibia (see Zhu et al. 2017: fig. 362D)	**6**
6	Conductor with posterior lobe (see Zhu et al. 2017: figs 361E, 362D)	***T.truculenta***
–	Conductor without lobe (see Zhu et al. 2017: fig. 363D, E)	**7**
7	Tip of conductor bifurcated (see Jiang et al. 2018: 89, fig. 24A–C	***T.rostrum***
–	Tip of conductor not bifurcated (see Zhu et al. 2017: figs 359D, E, 363D, E)	**7**
8	Tip of dorsal apophysis of conductor close to median apophysis of tegulum (see Zhu et al. 2017: fig. 363D, E)	***T.variegata***
–	Tip of dorsal apophysis of conductor separated with median apophysis of tegulum (see Zhu et al. 2017: fig. 359C–E)	***T.mopanensis***

**Females** (female of *T.makros* is unknown)

**Table:** 

1	Atrium located anteriorly	***T.defossa***
–	Atrium located posteriorly or medially	**2**
2	Epigynal teeth basally fused	***T.truculenta***
–	Epigynal teeth slightly separated	**3**
3	Spermathecal heads located antero-laterally	***T.lyrata***
–	Spermathecal heads located medially or posteriorly	**4**
4	Spermathecae coiled	***T.mopanensis***
–	Spermathecae sac-shaped, round or tube-shaped	**5**
5	Spermathecae separated by their width	***T.distalis***
–	Spermathecae, separated by less than radius	**6**
6	Spermathecal heads extending from median to anterior vulva	***T.eburniformis***
–	Spermathecal heads not extending median to anterior vulva	**7**
7	Copulatory ducts strongly expanded anteriorly	**8**
–	Copulatory ducts not anteriorly expanded	**9**
8	Spermathecal heads arising from median part of spermatheca	***T.variegata***
–	Spermathecal heads arising from postero-lateral part of spermathecae	***T.tautispina***
9	Spermathecae kidney-shaped, with a light constriction	***T.rostrum***
–	Spermathecae oval or tube-shaped with strong constriction	**10**
10	Spermathecal heads very long, tapering anteriorly	***T.jinggangensis* sp. nov.**
–	Spermathecal heads relatively short, not tapering anteriorly	**11**
11	Copulatory ducts extending along the lateral part of spermathecae; sspermathecae close to each other	***T.yanlingensis***
–	Copulatory ducts not extending along the lateral part of spermathecae; spermathecae, separated by less than each half width	***T.subyanlingensis* sp. nov.**

#### Tonsilla jinggangensis

Taxon classificationAnimaliaAraneaeAgelenidae

K. Liu & X. Xu sp. nov.

905323F6-ECC7-5E89-B53C-CA63EE5B2881

http://zoobank.org/8400223E-E24F-44FC-84D7-DA546F661BFC

Figures 1 , 2 , 3

##### Type material.

***Holotype*** ♀; China: Jiangxi Province, Ji’an City, Jinggangshan County Level City, Luofu Town, Pingtou Village, Jinggang Mountain National Nature Reserve, Changguling Forest Farm; 26°38'28"N, 114°14'6"E, 583 m; 5.X.2014; Ke-ke Liu et al. leg.

##### Etymology.

The specific name refers to the type locality; adjective.

##### Diagnosis.

**Figure 3.**
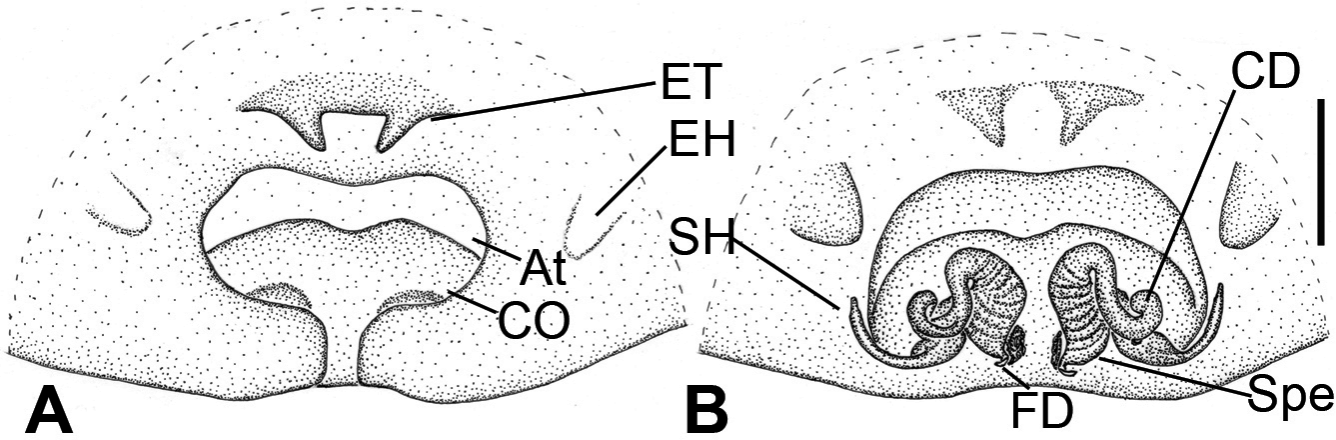
*Tonsillajinggangensis* sp. nov., female holotype **A** epigyne, ventral view **B** vulva, dorsal view. Scale bars: 0.5 mm. Abbreviations: At – atrium, CD – copulatory duct, CO – copulatory opening, EH – epigynal hood, ET – epigynal teeth, FD – fertilization ducts, SH – spermathecal heads, Spe – spermathecae.

The female of this species is similar to that of *T.yanlingensis* but differs by the long horn-shaped epigynal teeth (vs short, bell-shaped in *T.yanlingensis*), the widened posterior part of atrium (vs narrowed in *T.yanlingensis*) and the slender spermathecal heads (vs relatively short and curved in *T.yanlingensis*) (Figs 1C, D, 2, 3).

##### Description.

**Female.** Habitus as in Figure 1A, B. Total length 11.03. ***Carapace*** (Fig. 1A, B) 6.84 long, 3.70 wide, anteriorly narrowed to between 0.8- and 0.9-time maximum width of carapace. ***Eye*** (Fig. 1A) sizes and interdistances: AME 0.18; ALE 0.30; PME 0.20; PLE 0.24; AME–AME 0.18; AME–ALE 0.25; PME–PME 0.20; ALE–ALE 0.94; PME–PLE 0.38; PLE–PLE 1.38; ALE−PLE 0.18, AME−PME 0.21; AME–PLE 0.52. MOA: 0.62 long; 0.56 anterior width, 0.62 posterior width. ***Chelicerae*** (Fig. 1A, B) with 3 promarginal teeth (median largest) and 3 retromarginal teeth (median largest). ***Leg*** measurements (Fig. 1A, B): I 13.81 (3.82, 1.80, 2.91, 3.30, 1.98); II 12.24 (3.52, 1.76, 2.33, 2.88, 1.75); III 10.91 (3.23, 1.62, 1.83, 2.78, 1.45); IV 14.84 (4.22, 1.85, 3.11, 3.95, 1.71). ***Spination***: I fe 211, ti 022, mt 055; II fe 211, ti 033, mt 055; III fe 122, pa *111, ti 055, mt 855, ta 021; IV fe 101, pa *101, ti 055, mt 855, ta 022. Pedicel 0.40. ***Abdomen*** (Fig. 1A, B) 5.40 long, 3.26 wide.

Carapace brown. Chelicerae red brown. Endites, labium, and sternum yellow-brown. Legs yellow-brown. Abdomen dark brown, dorsally with 2 pairs of yellow-brown spots from antero-median to middle and 4 yellow-brown chevron-like stripes in posterior half.

***Epigyne*** (Figs 1C, D, 2, 3). Atrium deep, transverse, more than 2 times wider than long. Copulatory openings located at postero-lateral part of the atrium. Epigynal teeth long, horn-shaped, separated by its length, apex slightly convergent. Copulatory ducts slightly longer than spermathecae, originating posteriorly, extending forward along spermathecae and connected to anterior part of spermathecae. Spermathecae arched, with many constrictions, separated by less than radius of spermatheca. Spermathecal heads slender tube-shaped, posteriorly located, bent laterally. Fertilization ducts located at the posterior part of spermathecae.

##### Distribution.

Known only from the type locality in Jiangxi Province, China (Fig. 9).

##### Comments.

Although we have only the female of this species, we are convinced that it is not conspecific with *T.makros* a species known from Guizhou. The male of *T.makros* (6.20) is slightly larger than half of the female of *T.jinggangensis* sp. nov. (11.03). *Tonsilla* species seem to have a narrow distribution, except for *T.truculenta* from south China. *Tonsillajinggangensis* sp. nov. and *T.tautispina* from Jiangxi are more similar to species from Hunan, such as *T.lyrata* and *T.yanlingensis*, than species from Guizhou (*T.makros*, *T.mopanensis*). Hence, the male palp of this species may have a stout patellar apophysis.

#### Tonsilla subyanlingensis

Taxon classificationAnimaliaAraneaeAgelenidae

K. Liu & X. Xu sp. nov.

1534F6B8-7D78-529F-B6A4-C03A07DAF08A

http://zoobank.org/64760314-489A-4A5C-AFA3-3D857B92ED14

**Figure 4.**
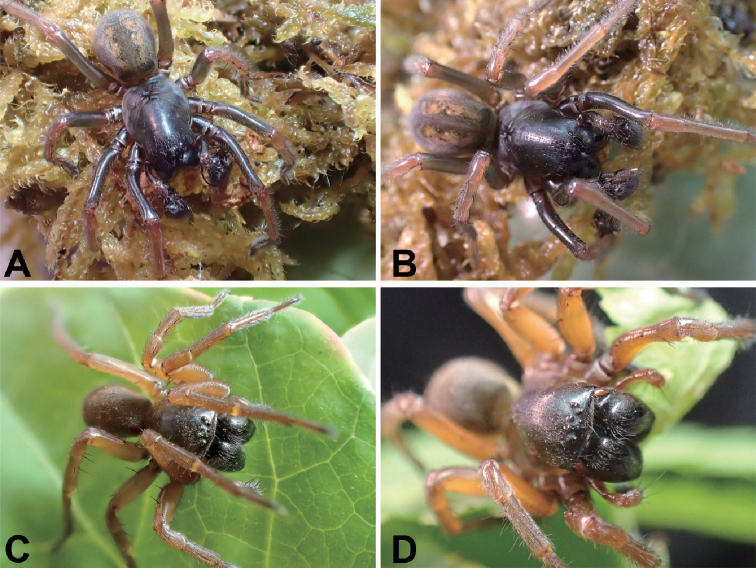
Photographs of living specimens of *Tonsillasubyanlingensis* sp. nov. from Jinggang Mountain. **A, B** male **C, D** female.

**Figure 5.**
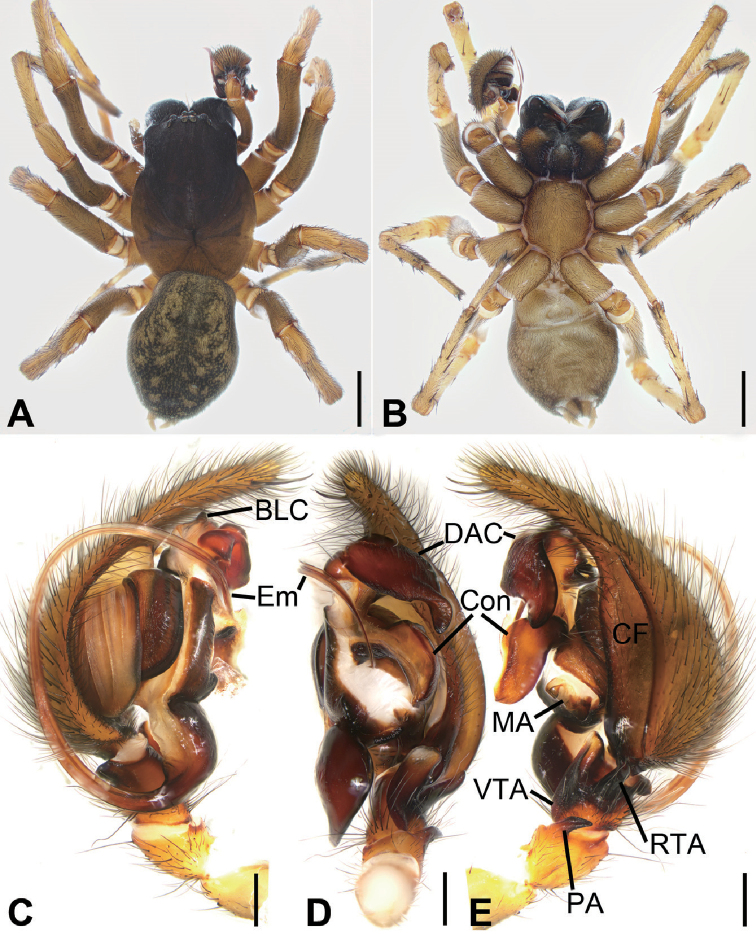
*Tonsillasubyanlingensis* sp. nov., male holotype **A** habitus, dorsal view **B** same, ventral view **C** palp, prolateral view **D** same, ventral view **E** same, retrolateral view. Scale bars: 2 mm (**A, B**), 1 mm (**C–E**). Abbreviations: BLC – basal lamella of conductor, CF – cymbial furrow, Con – conductor, DAC – dorsal apophysis of conductor, Em – embolus, ET – epigynal teeth, MA – median apophysis, RTA – retrolateral tibial apophysis, PA – patellar apophysis, VTA – ventrolateral tibial apophysis.

**Figure 6.**
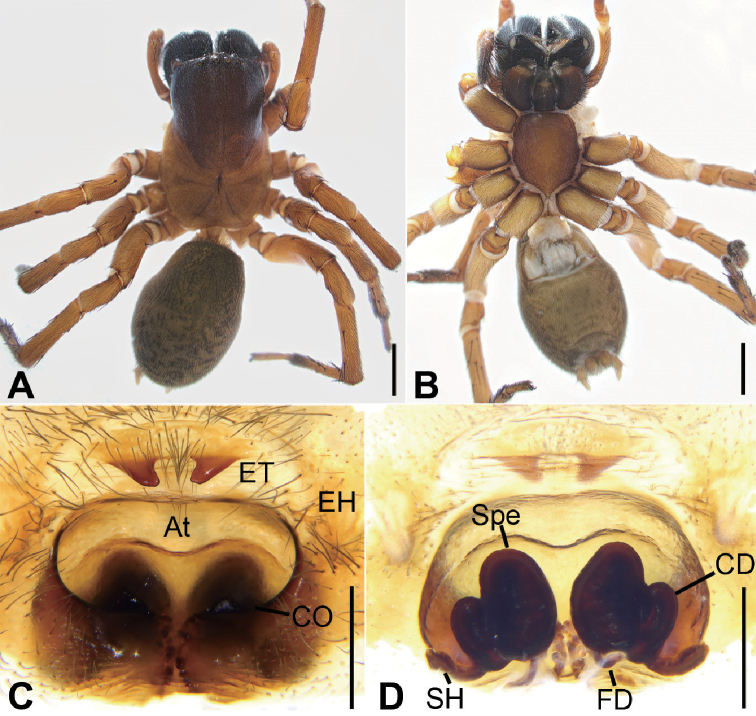
*Tonsillasubyanlingensis* sp. nov., female paratype **A** habitus, dorsal view **B** same, ventral view **C** epigyne, ventral view **D** vulva, dorsal view. Scale bars: 2 mm (**A, B**), 0.5 mm (**C, D**). Abbreviations: At – atrium, CD – copulatory duct, CO – copulatory opening, EH – epigynal hood, ET – epigynal teeth, FD – fertilization ducts, SH – spermathecal heads, Spe – spermathecae.

**Figure 7.**
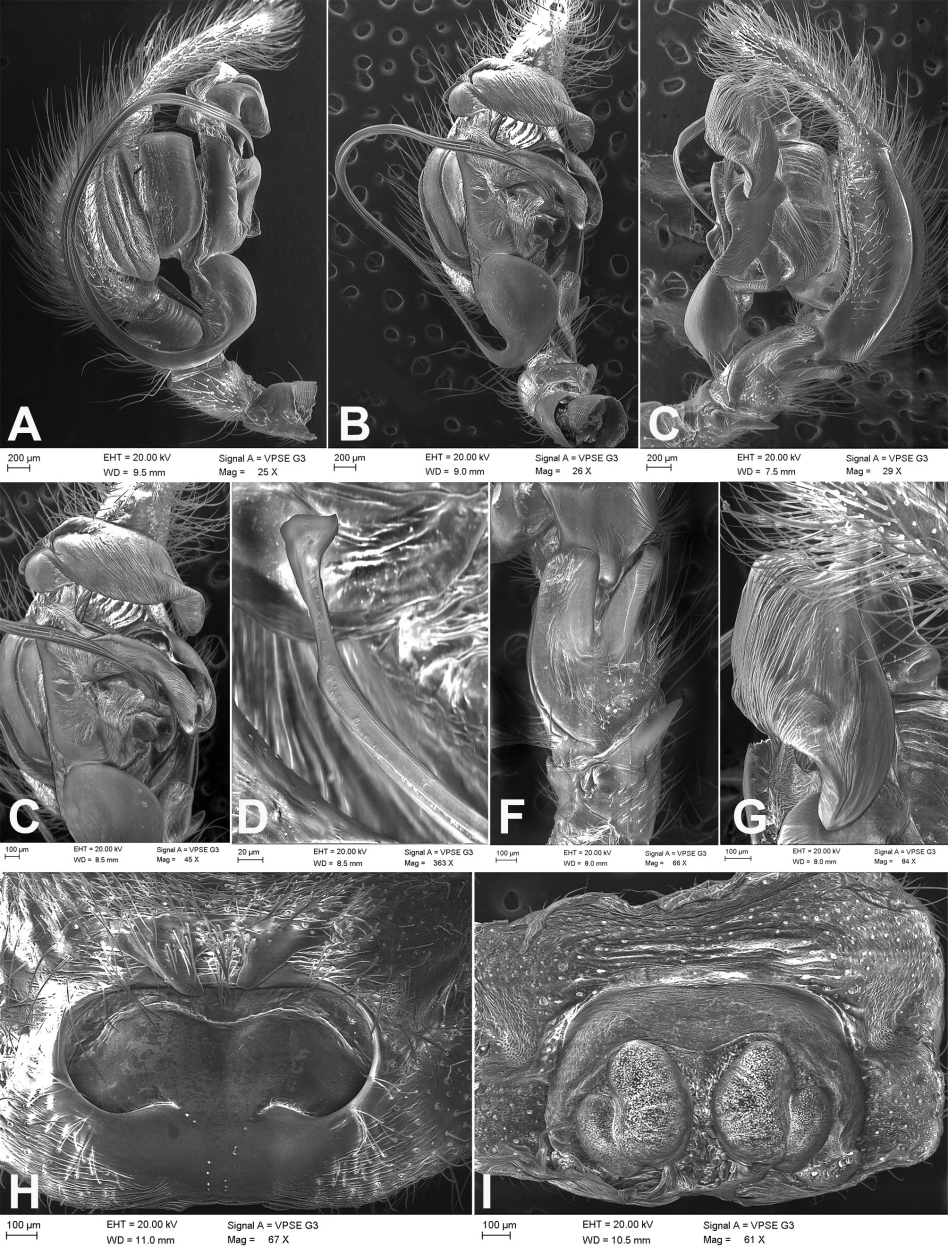
SEM images of *Tonsillasubyanlingensis* sp. nov., male holotype and female paratype **A** palp, prolateral view **B** same, ventral view **C** same, detail of conductors, ventral view **D** same, detail of embolus, ventral view **E** same, retrolateral view **F** same, detail of patellar apophysis, retrolateral tibial apophysis and lateral tibial apophysis, retrolateral view **G** same, detail of conductor dorsal apophysis, retrolateral view **H** epigyne, dorsal view **I** vulva, ventral view.

**Figure 8.**
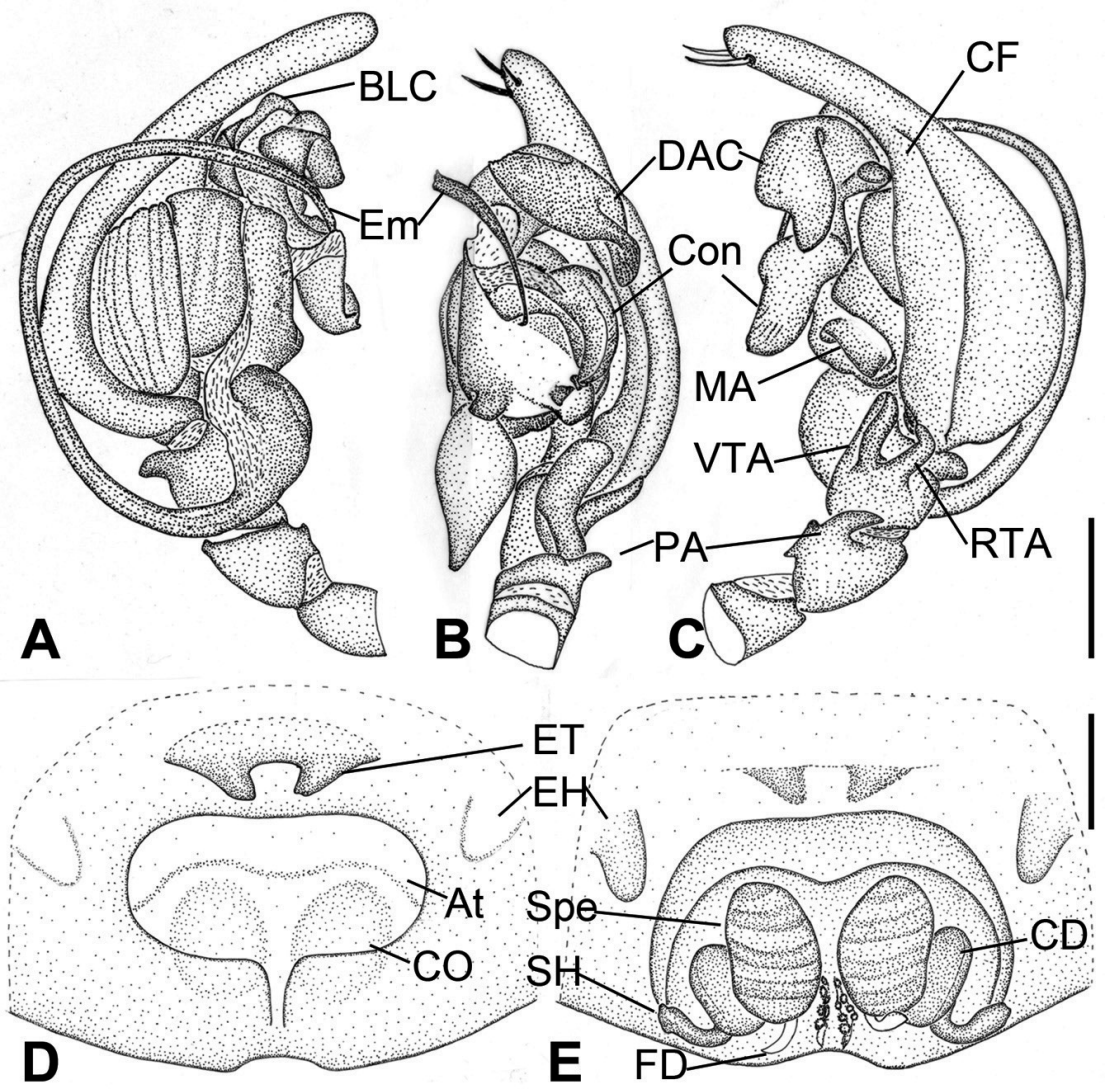
*Tonsillasubyanlingensis* sp. nov., male holotype and female paratype **A** male palp, prolateral view **B** same, ventral view **C** same, retrolateral view **D** epigyne, ventral view **E** vulva, dorsal view. Scale bars: 1 mm. Abbreviations: At – atrium, BLC – basal lamella of conductor, CD – copulatory duct, CF – cymbial furrow, CO – copulatory opening, Con – conductor, DAC –dorsal apophysis of conductor, EH – epigynal hood, Em – embolus, ET – epigynal teeth, FD – fertilization ducts, MA – median apophysis, PA – patellar apophysis, RTA – retrolateral tibial apophysis, SH – spermathecal heads, Spe – spermathecae, VTA – ventrolateral tibial apophysis.

Figures 4 , 5 , 6 , 7 , 8

##### Type material.

***Holotype*** ♂; China: Jiangxi Province, Ji’an City, Jinggangshan County Level City, Ciping Town, Wuzhi Peak Scenic Spot; 26°31'59.07"N, 114°08'28.47"E, 735 m; 2.X.2018; Ke-ke Liu et al. leg. ***Paratypes***: 2 ♀; same data as holotype; 1 ♀; same locality, Dajing Village; 26°33'50.4"N, 114°07'26.4"E, 930 m; 19.X.2014; Ke-ke Liu et al. leg.; 1 ♀; same locality; 26°34'12.89"N, 114°07'41.87"E, 950 m; 30.IX.2018; Ke-ke Liu et al. leg.; 1 ♀; same locality, Jingzhushan Scenic Spot; 26°31'33.37"N, 114°06'30.34"E, 786 m; 1.X.2018; Ke-ke Liu et al. leg.

##### Etymology.

The specific name refers to its similarity to *T.yanlingensis* (Zhang, Yin & Kim, 2000); adjective.

##### Diagnosis.

Females of the new species closely resemble *T.yanlingensis* by the heart-shaped, large atrium and wide epigynal teeth, but can be distinguished by the spermathecae separated by less than 1/5 of their width (vs touching each other in *T.yanlingensis*), long and broad copulatory ducts along with the spermathecae (vs very short in *T.yanlingensis*), the slightly procurved spermathecal heads located at posterior part of spermatheca (Figs 5C, D, 7H, I) (vs strong procurved spermathecal heads located at mid part of spermatheca in vulva), and the spermathecae slightly separated by less than 1/5 of their width (vs touching each other) (Wang and Yin 1992; Zhu et al. 2017). The male of this species is similar to that of *T.mopanensis* and *T.truculenta* in having a long, broad, and furrowed basal lamella of conductor, but can be separated by the patellar apophysis which is relatively shorter than patellar (vs as long as patellar apophysis in *T.mopanensis* or longer in *T.truculenta*) and the conductor with a long, broad curved dorsal apophysis (Figs 5A–C, 7A–G, 8A–C) (vs long, narrowed in *T.mopanensis*; short, strong in *T.truculenta*).

##### Description.

**Male** (Holotype). Habitus as in Figures 4A, B, 5A, B. Total length 11.25. ***Carapace*** (Fig. 5A) 6.01 long, 4.44 wide, anteriorly narrowed to between 0.6 and 0.7 its maximum width. ***Eye*** sizes and interdistances: AME 0.20; ALE 0.25; PME 0.24; PLE 0.25; AME–AME 0.10; AME–ALE 0.18; PME–PME 0.08; ALE–ALE 0.81; PME–PLE 0.36; PLE–PLE 1.16; ALE−PLE 0.10, AME−PME 0.16; AME–PLE 0.45. MOA: 0.60 long; 0.49 front width, 0.50 back width. ***Chelicerae*** (Fig. 5B) with 2 promarginal teeth (proximal smaller) and 2 retromarginal teeth (proximal larger). ***Leg*** (Fig. 5A, B) measurements: I 17.84 (4.88, 1.95, 4.46, 4.30, 2.25); II 15.78 (4.25, 1.90, 3.74, 3.74, 2.15); III 13.36 (3.62, 1.81, 2.82, 3.40, 1.71); IV 17.27 (4.87, 1.92, 3.69, 4.77, 2.02). ***Spination***: I fe 120, pa 001, ti 055, mt 033, II fe 000, ti 044, mt 033,. Femur I with 6 ventral cusps. Leg measurements (Fig. 5A, B): I 13.15 (4.17, 1.85, 3.26, 2.54, 1.33); II 12.90 (3.62, 1.18, 2.90, 2.83, 1.71); III 11.00 (3.01, 1.72, 2.14, 2.75, 1.38); IV 14.12 (3.84, 1.87 3.45, 3.70, 1.32). Spination: I fe 210, ti 043, mt 055; II fe 210, ti 043, mt 065; III fe 121, pa 011, ti 433, mt 655, ta 011; IV fe 100, ti 342, mt 753, ta 012. Pedicel 0.32. ***Abdomen*** (Fig. 5A, B) 5.24 long, 3.68 wide.

Carapace dark brown. Chelicerae dark brown. Endites and labium dark yellow-brown. Sternum and legs yellow-brown. Abdomen dark brown with 5 pairs of yellow-brown spots on posterior half.

***Palp*** (Figs 5C–E, 7A–G, 8A–C). Femur more than 2 times longer than patella. Patellar apophysis slightly shorter than patella, thumb shaped. Tibia with wide ventrolateral apophysis and long retrolateral apophysis, the former extending beyond tibia, strongly sclerotized; the later slightly shorter than ventrolateral, and twice thinner, apex bent ventrally to the tip of ventrolateral apophysis, forming a right angle with its axis. Cymbium, approximately 3 times longer than wide, cymbial furrow less than 2/3 of the cymbial length, approximately 1/3 of cymbial width in retrolateral view. Median apophysis spoon-shaped, located near the base of embolus; conductor, slightly curved, with a long, broad and furrowed basal lamella and a large, twisted, sclerotized dorsal apophysis; embolus long and broad, originates at 6 o’clock position, coiled around the margin of cymbium and posteriorly embedded in the furrow of conductor.

**Female** (Paratype). Habitus as in Figures 4C, D, 6A, B. Total length 13.21. ***Carapace*** (Fig. 6A) 6.66 long, 4.33 wide, anteriorly narrowed to between 0.7 and 0.8 its maximum width. ***Eye*** sizes and interdistances: AME 0.20; ALE 0.24; PME 0.22; PLE 0.24; AME–AME 0.10; AME–ALE 0.18; PME–PME 0.20; ALE–ALE 0.92; PME–PLE 0.41; PLE–PLE 1.46; ALE−PLE 0.14, AME−PME 0.16; AME–PLE 0.45. MOA: 0.68 long; 0.60 front width, 0.70 back width. ***Chelicerae*** (Fig. 6A, B) with 3 promarginal teeth (proximal smallest, median largest) and 3 retromarginal teeth (proximal largest). ***Leg*** measurements (Fig. 6A, B): I 13.15 (4.17, 1.85, 3.26, 2.54, 1.33); II 12.90 (3.62, 1.18, 2.90, 2.83, 1.71); III 11.00 (3.01, 1.72, 2.14, 2.75, 1.38); IV 14.12 (3.84, 1.87 3.45, 3.70, 1.32). ***Spination***: I fe 402, ti 004, mt 044; II fe 122, pa 011, ti 035, mt 065; III fe 122, pa 111, ti 055, mt 622, ta 011; IV fe 122, pa 011, ti 055, mt 866, ta 011. Pedicel 0.46. ***Abdomen*** (Fig. 6B, C) 6.10 long, 4.01 wide.

Lighter than male. Abdomen, dorsally with four indistinct yellow-brown chevron-like stripes on posterior half.

***Epigyne*** (Figs 6C, D, 7H, I, 8D, E). Atrium with a transverse depression, broad, more than 2 times longer than its length, heart-shaped, anterior margin near the apex of teeth, posterior part relatively broad. Copulatory openings located at postero-lateral of the atrium. Epigynal teeth flat, separated by less than their length, apex slightly converging. Copulatory ducts, originating laterally, extending forward along spermathecae, then back, but located at lateral part of spermathecae. Spermathecae egg-shaped, clearly separated by less than 1/5 their width. Spermathecal heads relatively broad, short, posteriorly located, curved laterally. Fertilization ducts located at the posterior part of the spermathecae.

##### Distribution.

**Figure 9.**
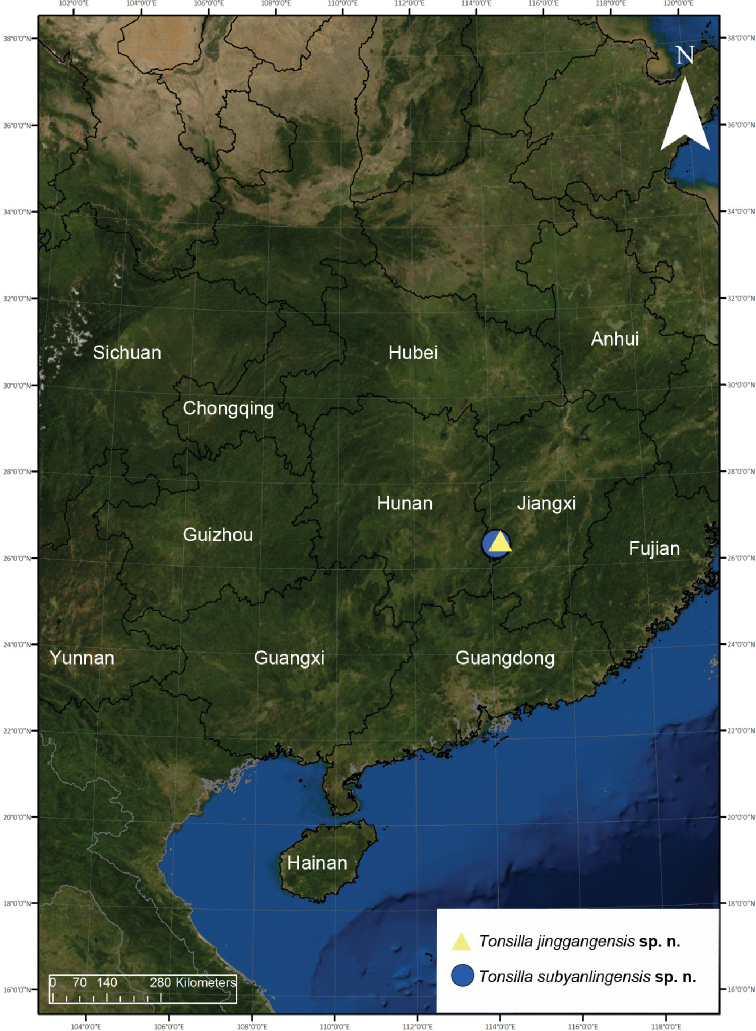
Type localities of *Tonsillajinggangensis* sp. nov. and *T.subyanlingensis* sp. nov.

Known only from the type locality in Jiangxi Province, China (Fig. 9).

## Supplementary Material

XML Treatment for Tonsilla

XML Treatment for Tonsilla jinggangensis

XML Treatment for Tonsilla subyanlingensis
